# The adaptor protein SH2B1β reduces hydrogen peroxide-induced cell death in PC12 cells and hippocampal neurons

**DOI:** 10.1186/1750-2187-5-17

**Published:** 2010-09-27

**Authors:** Wan-Chen Lu, Chien-Jen Chen, Hui-Chien Hsu, Hsin-Ling Hsu, Linyi Chen

**Affiliations:** 1 Institute of Molecular Medicine, National Tsing Hua University, Hsinchu, Taiwan; 2 Department of Life Science, National Tsing Hua University, Hsinchu, Taiwan; 3 Brain Research Center, National Tsing Hua University, Hsinchu, Taiwan; 4 Division of Molecular and Genomic Medicine, National Health Research Institutes, Miaoli County, Taiwan

## Abstract

**Background:**

SH2B1β is a signaling adaptor protein that has been shown to promote neuronal differentiation in PC12 cells and is necessary for the survival of sympathetic neurons. However, the mechanism by which SH2B1β may influence cell survival is not known.

**Results:**

In this study, we investigated the role of SH2B1β in oxidative stress-induced cell death. Our results suggest that overexpressing SH2B1β reduced H_2_O_2_-induced, caspase 3-dependent apoptosis in PC12 cells and hippocampal neurons. In response to H_2_O_2_, overexpressing SH2B1β enhanced PI3K (phosphatidylinositol 3-kinas)-AKT (protein kinase B) and MEK (MAPK/ERK kinase)-extracellular-signal regulated kinases 1 and 2 (ERK1/2) signaling pathways. We further demonstrated that SH2B1β was able to reduce H_2_O_2_-induced nuclear localization of FoxO1 and 3a transcription factors, which lie downstream of PI3K-AKT and MEK-ERK1/2 pathways. Moreover, overexpressing SH2B1β reduced the expression of Fas ligand (FasL), one of the target genes of FoxOs.

**Conclusions:**

Overexpressing the adaptor protein SH2B1β enhanced H_2_O_2_-induced PI3K-AKT and MEK-ERK1/2 signaling, reduced nucleus-localized FoxOs and the expression of a pro-apoptotic gene, FasL.

## Introduction

Oxidative stress resulting from overload of toxic reactive oxygen species (ROS) is common in the etiology of human diseases. It has been implicated in various neurodegenerative diseases, including Alzheimer's disease, Parkinson's disease, and Huntington's disease [1-4]. It also contributes to acute damage resulting from hypoxic-reperfusion conditions after trauma or stroke [5,6]. The accumulation of ROS, such as hydrogen peroxide (H_2_O_2_), leads to various forms of reversible and irreversible oxidative modification of proteins, lipids and DNA, accounting for cellular damage [7]. Depending on the extent of oxidative stress, it can induce proliferation, growth arrest, senescence, apoptosis (programmed cell death) or necrosis [8-11].

A number of signaling pathways are evolved to protect cells from ROS-induced damages, including phosphatidylinositol 3-kinase (PI3K)-AKT pathway, mitogen-activated protein kinases (MAPKs) pathways, and phospholipase Cγ (PLCγ) signaling [12-20]. PI3K-AKT pathway predominantly acts to promote cell survival. The three family members of MAPKs are identified as being sensitive to oxidative stress. They are extracellular-signal regulated kinase 1/2 (ERK1/2), c-Jun N-terminal kinase (JNK), and p38MAPK. Controversial reports implicating the influence of oxidative stress-induced MAPK activation on both cell survival and death are more complicated than one has anticipated [21-30]. In most cases, MEK-ERK1/2, similar to PI3K-AKT pathway, promotes cell survival in response to oxidative stress.

SH2B1 is a signaling adaptor protein that belongs to SH2B family, including SH2B1, SH2B2 (APS) and SH2B3 (Lnk) [31,32]. SH2B1 has been implicated in signaling pathways initiated by several receptor tyrosine kinases, including growth hormone, nerve growth factor (NGF), insulin, insulin-like growth factor 1, brain-derived neurotrophic factor, glial-derived neurotrophic factor, platelet-derived growth factor, and fibroblast growth factor 1 [31,33-41]. Four isoforms have been identified for SH2B1 --- α, β, γ and δ [33]. Previous studies demonstrate that SH2B1 plays an essential role in neuronal differentiation of PC12 cells, a well-established neuronal model [37,39,41,42]. SH2B1β also supports axonal growth of sympathetic neurons and is required for the survival of neonatal sympathetic neurons [37]. Moreover, SH2B1β acts as a positive mediator of NGF-mediated activation of AKT/Forkhead pathway by affecting the subcellular distribution of FoxO1 and 3a [43]. Forkhead transcription factors comprise more than 100 structurally related members that share a conserved forkhead domain (FKH) and a 100-residue DNA-binding domain. They have been named Fox (forkhead box) transcription factors [44]. Mammalian FoxO proteins (FoxO1, 3, 4 and 6) belong to O (other) class of the Fox superfamily. The nucleus-localized FoxOs are known to induce the expression of pro-apoptotic genes, such as FasL (Fas ligand) [45]. Therefore, inactivating FoxOs prevents their entry to the nucleus and triggering apoptosis. AKT is known to phosphorylate FoxOs and thus reduces their nuclear localization [46-49]. MAPKs have also been reported to phosphorylate FoxOs [50-52]. The fact that overexpressing SH2B1β shifts the steady-state distribution of FoxO1 in PC12 cells [43] raises a possibility that SH2B1β may affect cell survival through FoxO family members. To understand how SH2B1β may regulate cell survival/death, cells were challenged with oxidative stress and the effect of SH2B1β was examined. In this study, we investigated the role of SH2B1β in oxidative stress-induced cell death, signaling, FoxOs distribution and their target gene expression.

## Results

### Overexpressing SH2B1β reduces hydrogen peroxide-induced cell death in PC12 cells

**Figure 1:**
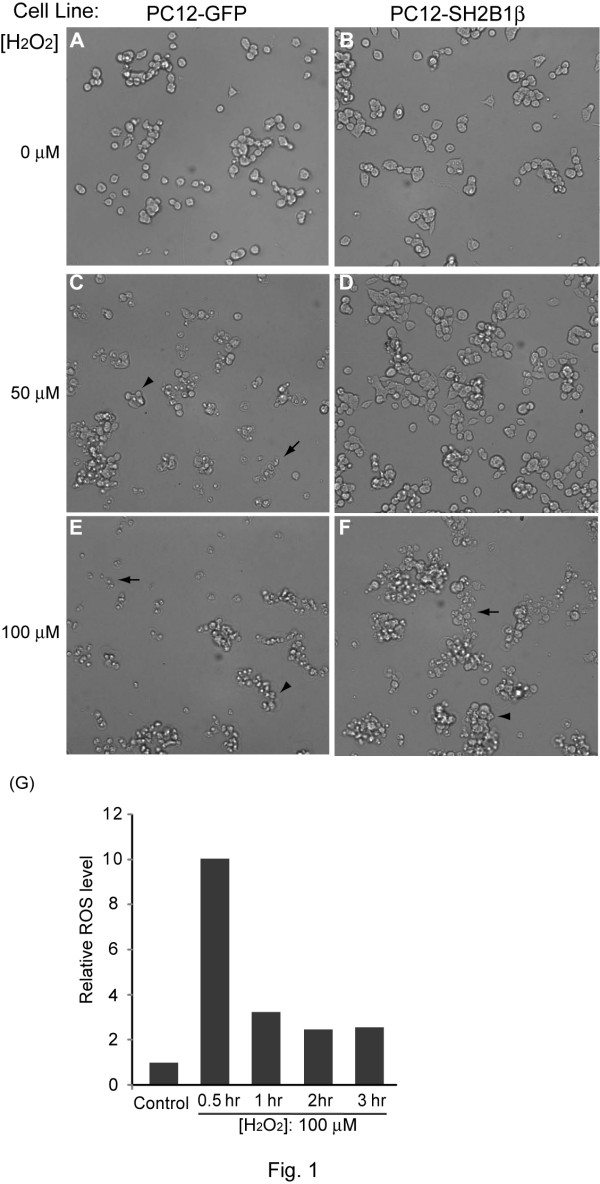
**Cell survival of H_2_O_2_-treated PC12-GFP and PC12-SH2B1β cells**. PC12-GFP cells (A, C, E) and PC12-SH2B1β cells (B, D, F) were treated with 0 (A, B), 50 (C, D), or 100 (E, F) μM H_2_O_2 _for 24 h. Representative images of live cells were shown. (G) Parental PC12 cells were treated with 100 μM H_2_O_2 _for the indicated time points. ROS levels were measured using DHE dye as described in "Materials and Methods". Arrows point to dying cells. Arrowheads point to live cells.

**Figure 2:**
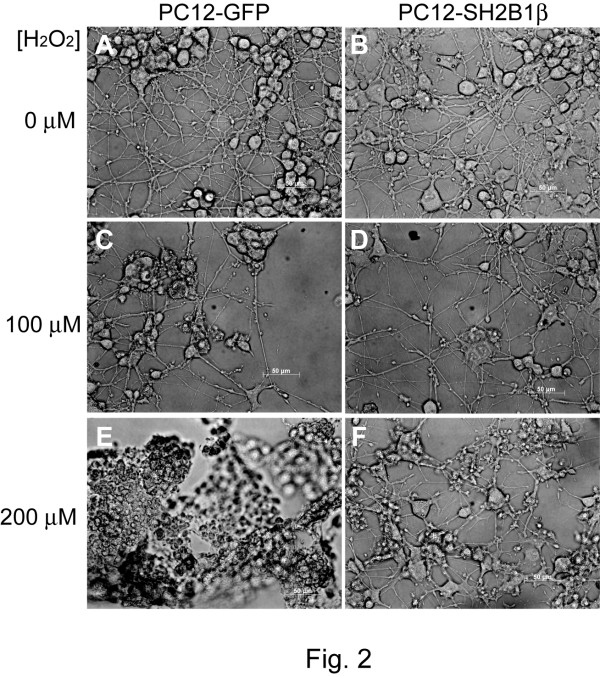
**H_2_O_2 _-induced cell death and neurite retraction in differentiated PC12-GFP and PC12-SH2B1β cells**. PC12-GFP cells (A, C, E) and PC12-SH2B1β cells (B, D, F) were differentiated using 50 ng/ml NGF for 7 days. After overnight incubation in serum-free medium containing 50 ng/ml NGF, both cell lines were treated with 0, 100 or 200 μM H_2_O_2 _for 24 h. Representative images were shown (400× magnification).

**Figure 3:**
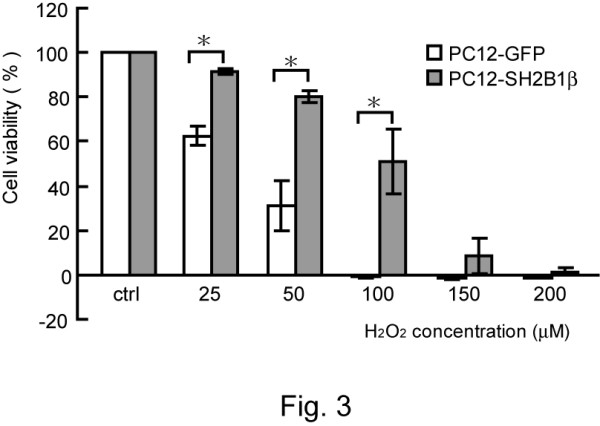
**Cell viability of PC12-GFP and PC12-SH2B1β cells treated with H_2_O_2_**. PC12-GFP or PC12-SH2B1β cells were treated with the indicated concentrations of H_2_O_2 _for 24 h. Cell viability was measured by MTT assays as described in "Materials and Methods". Values are expressed as % of control (untreated samples) for each experiment, with the values obtained for untreated cells taken as 100%. Values are expressed as mean ± S.E. from three independent experiments. The asterisks represent significant differences compared with control cells (*, p < 0.05).

To determine whether SH2B1β affects oxidative stress-induced cell death, PC12 cells stably expressing GFP (PC12-GFP cell line) or GFP-SH2B1β (PC12-SH2B1β cell line) were treated without (Figure 1A, B) or with (Figure 1C, D, E, F) H_2_O_2_. With increasing concentration of H_2_O_2_, both cell lines showed increased cell death. Notably, PC12-SH2B1β cells showed less cell death compared to PC12-GFP cells. To verify that H_2_O_2 _treatment effectively increased cellular oxidative stress, an oxidation indicator dye, dihydroethidine (DHE), was used to monitor cellular oxidation. As shown in Figure 1G, oxidative stress was increased within 30 min of 100 μM H_2_O_2 _treatment. The elevated ROS was reduced afterwards, likely through cellular reduction, and remained higher than basal level for at least 3 h. This dosage of H_2_O_2 _also resulted in death of primary culture of hippocampal neurons (Additional file 1). The protective effect of overexpressing SH2B1β in H_2_O_2_-treated differentiated PC12 cells was also examined. H_2_O_2 _treatment induced retraction of neurites as well as death of differentiated PC12 cells. Similarly, differentiated PC12-SH2B1β cells showed less cell death compared to differentiated PC12-GFP cells. These results suggest that overexpressing SH2B1β reduces H_2_O_2_-induced cell death in both undifferentiated and differentiated PC12 cells (Figure 2). To quantify cell viability, MTT assays were used to assess H_2_O_2_-induced cell death in PC12 cells. In all H_2_O_2 _concentrations tested, cell survival was higher in PC12-SH2B1β cells compared to PC12-GFP cells (Figure 3). For instance, as most of PC12-GFP cells underwent dramatic cell death when treated with 100 μM H_2_O_2 _for 24 h, PC12-SH2B1β remained nearly 50% survival rate (Figure 3).

### H_2_O_2 _induces caspase 3-dependent cell death in PC12 cells

**Figure 4:**
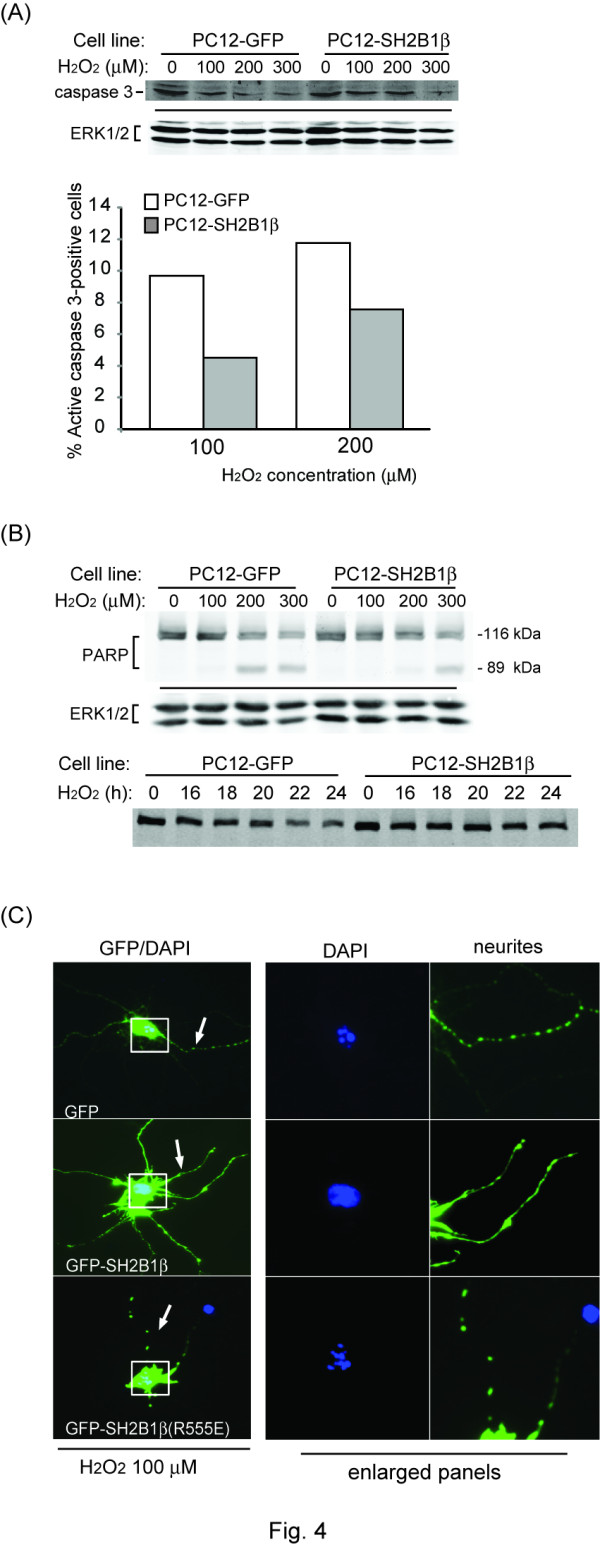
**H_2_O_2 _induces caspase 3-dependent apoptosis in PC12-GFP and PC12-SH2B1β cells**. PC12-GFP and PC12-SH2B1β cells were treated with 0, 100, 200, 300 μM H_2_O_2 _for 18 h. Equal amounts of proteins from the lysates were resolved with SDS-PAGE and immunoblotted with (A) anti-caspase 3 (upper panel), or (B) anti-PARP antibody. ERK levels were used as loading controls. For % cells with anti-active caspase 3 staining, cells were treated with 100 or 200 μM H_2_O_2 _for 18 h and then subjected to immunofluorescence staining using anti-active caspase 3 antibody (A, lower panel). Percentages of active caspase 3-positive cells were counted from 145-211 cells/condition. (C) Hippocampal neurons from E18 embryos were transiently transfected with GFP, GFP-SH2B1β or GFP-SH2B1β(R555E) and then treated with H_2_O_2 _for 18 h. Cells were then subjected to immunofluorescence staining with DAPI (shown in blue) to mark the nucleus. Green fluorescence (GFP) showed the transfected cells. Boxes mark the nucleus and arrows point to the neurites. Enlarged images of the nucleus and neurites are shown on the right panels.

Low level of oxidative stress has been suggested to lead to apoptosis while high level of oxidative stress leads to apoptosis and necrosis [8,9,53]. In the present study, relatively low concentrations of H_2_O_2 _were used to more closely reflect the physiological stress [8]. During early apoptosis, phospholipids phosphatidylserine (PS) from the inner leaflet is translocated to the outer leaflet of the plasma membrane allowing for Annexin V binding. Thus, detecting the relative amount of Annexin V binding was measured to determine whether H_2_O_2 _induces apoptosis in PC12 cells. The relative Annexin V binding was increased in response to H_2_O_2 _treatment suggesting that concentrations of H_2_O_2 _used in this study induced apoptosis (Additional file 2). The processes of apoptosis could be caspase-dependent or caspase-independent (e.g. necroptosis) [54-61]. To further determine whether H_2_O_2 _induces caspase 3-dependent apoptosis and whether overexpressing SH2B1β affects caspase 3 activity, PC12-GFP and PC12-SH2B1β cells were treated with H_2_O_2 _and the level of full length caspase 3 was determined via western blotting. In response to H_2_O_2_, full length caspase 3 was reduced, resulting from activation and cleavage of caspase 3 (Figure 4A, upper panel). The relative amount of full length caspase 3 was higher in PC12-SH2B1β cells compared to PC12-GFP cells. The population of active caspase 3-positive cells was also lower in PC12-SH2B1β cells than in PC12-GFP cells (Fig. 4A, lower panel). Along this line, the relative amount of poly (ADP-ribose) polymerase (PARP), a substrate of caspase 3, was determined in PC12-GFP and PC12-SH2B1β cells to reflect the relative activity of caspase 3. The relative level of full length PARP was higher in PC12-SH2B1β cells compared to PC12-GFP cells and the reduction of full length PARP was more dramatic after 22 h of H_2_O_2 _challenge in PC12-GFP cells (Figure 4B). These data suggest that H_2_O_2 _induces caspase 3-dependent apoptosis in PC12 cells and overexpressing SH2B1β reduces the activity of caspase 3 and thus PARP cleavage. Similarly, the active caspase 3 was more prominent in hippocampal neurons overexpressing GFP than those overexpressing GFP-SH2B1β. In contrast, hippocampal neurons overexpressing the dominant negative mutant of SH2B1β, GFP-SH2B1β(R555E), were more susceptible to H_2_O_2_, leading to more caspase 3 cleavage compared to control cells (Additional file 3). Another phenotype of cells undergoing apoptosis is nuclear condensation. Hippocampal neurons subjected to H_2_O_2 _treatment showed obvious neurite retraction, beaded dendrites and condensation of the nucleus. As majority of neurons overexpressing GFP-SH2B1β showed intact nucleus, neurons that expressing GFP or GFP-SH2B1β(R555E) showed fragmented nucleus (Figure 4C). Together, these data demonstrate that SH2B1β reduces H_2_O_2_-induced caspase 3-dependent apoptosis in both PC12 cells and hippocampal neurons.

### Overexpressing SH2B1β enhances H_2_O_2_-induced phosphorylation of AKT and ERK1/2

**Figure 5:**
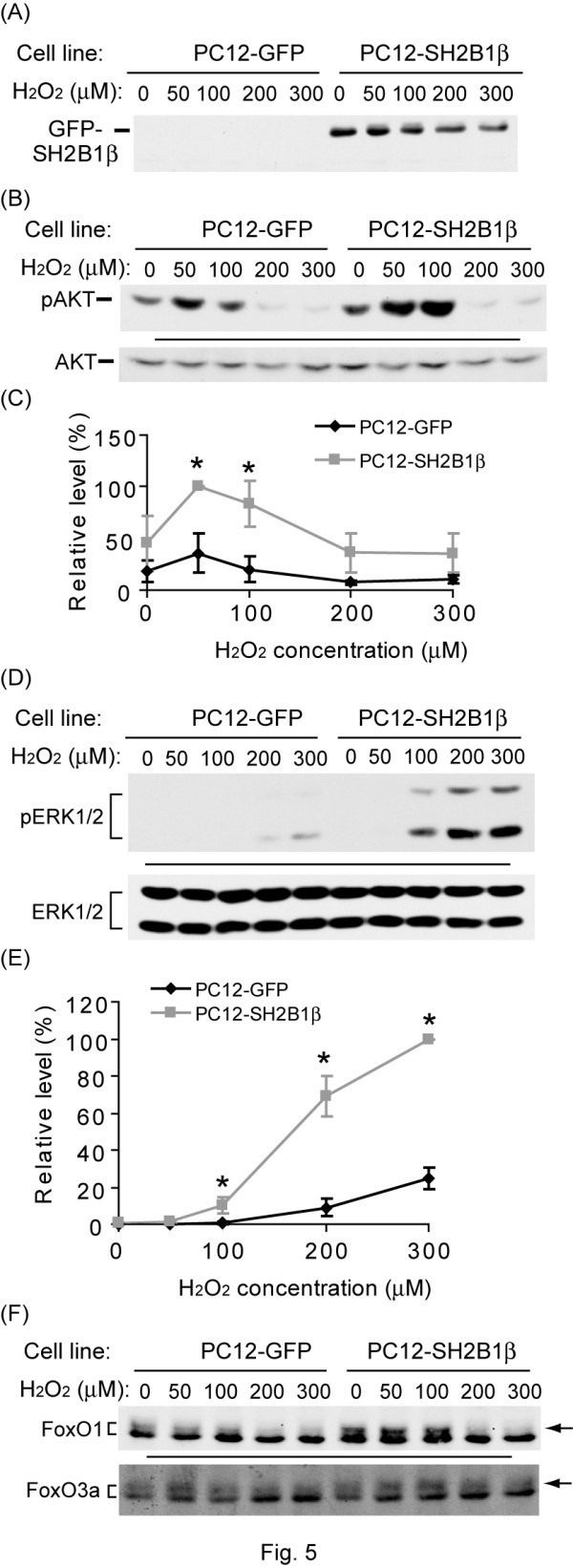
**SH2B1β enhances the phosphorylation levels of AKT, ERK1/2 and FoxOs**. PC12-GFP and PC12-SH2B1β cells were incubated in serum-free medium overnight followed by H_2_O_2 _stimulation with the indicated concentrations for 10 min. Equal amounts of proteins from the lysates were resolved via SDS-PAGE and immunoblotted with (A) anti-GFP; (B) anti-pAKT(Ser473) and anti-AKT; (D) anti-pERK1/2 and anti-ERK1/2; (F) anti-FoxO1 and anti-FoxO3a antibodies. Representative blots were shown. (C) The quantified results of pAKT in PC12-GFP and PC12-SH2B1β cells were shown. The levels of pAKT were normalized to levels of AKT at each condition. The relative level of pAKT at 50 μM of H_2_O_2 _in PC12-SH2B1β cells was defined as 100% in each experiment and others were normalized to this value. (E) The levels of pERK1/2 were normalized to total ERK1/2 levels, the relative level of pERK1/2 at 300 μM in PC12-SH2B1β cells was defined as 100% for each experiment and others were normalized to this value. Data are expressed as mean ± S.E. from three (for AKT) or five (for ERK1/2) independent experiments. Arrows point to the phospho-FoxO1 and 3a.

To investigate the mechanisms by which SH2B1β protects cells from oxidative stress, the effect of overexpressing SH2B1β on H_2_O_2_-induced cellular signaling was examined. Figure 5A showed that GFP-SH2B1β was overexpressed in PC12-SH2B1β cells but not in PC12-GFP cells. In PC12-GFP cells, phosphorylation of AKT (pAKT) was induced in response to 50 μM H_2_O_2. _On the other hand, overexpressing SH2B1β significantly enhanced the levels of pAKT in response to 50 and 100 μM H_2_O_2 _and, as H_2_O_2 _concentration increased, pAKT decreased (Figure 5B, C). Overall, the levels of pAKT were higher in PC12-SH2B1β than in PC12-GFP cells. Different from pAKT signal, phosphorylation of ERK1/2 (pERK1/2) was induced by H_2_O_2 _concentration higher than 200 μM in PC12-GFP cells and 100 μM in PC12-SH2B1β cells. H_2_O_2_-induced pERK1/2 was much more enhanced in PC12-SH2B1β cells compared to PC12-GFP cells (Figure 5D). The quantified results are shown in Figure 5E. Together, these results suggest that SH2B1β enhances H_2_O_2_-induced PI3K-AKT and MEK-ERK1/2 signaling.

### SH2B1β enhances phosphorylation of FoxOs, reduces their nuclear localization and target gene expression

FoxO transcription factors are known downstream effectors of AKT [46,51,62,63]. They have also been reported to be substrates of pERK1/2, p38MAPK and pJNK [23,50,64]. Since their subcellular distribution is controlled by phosphorylation, the downstream gene expression is likely affected by their phosphorylation status. As SH2B1β enhanced both pAKT and pERK1/2 levels, the phosphorylations of FoxO1 and 3a were examined. As in Figure 5F, phosphorylated FoxO1 and 3a were slightly increased in response to 50 μM H_2_O_2 _and then decreased when treated with 100 μM H_2_O_2 _and above. The extents of FoxO1 and 3a phosphorylation were more prominent in PC12-SH2B1β cells than those in PC12-GFP cells.

**Figure 6:**
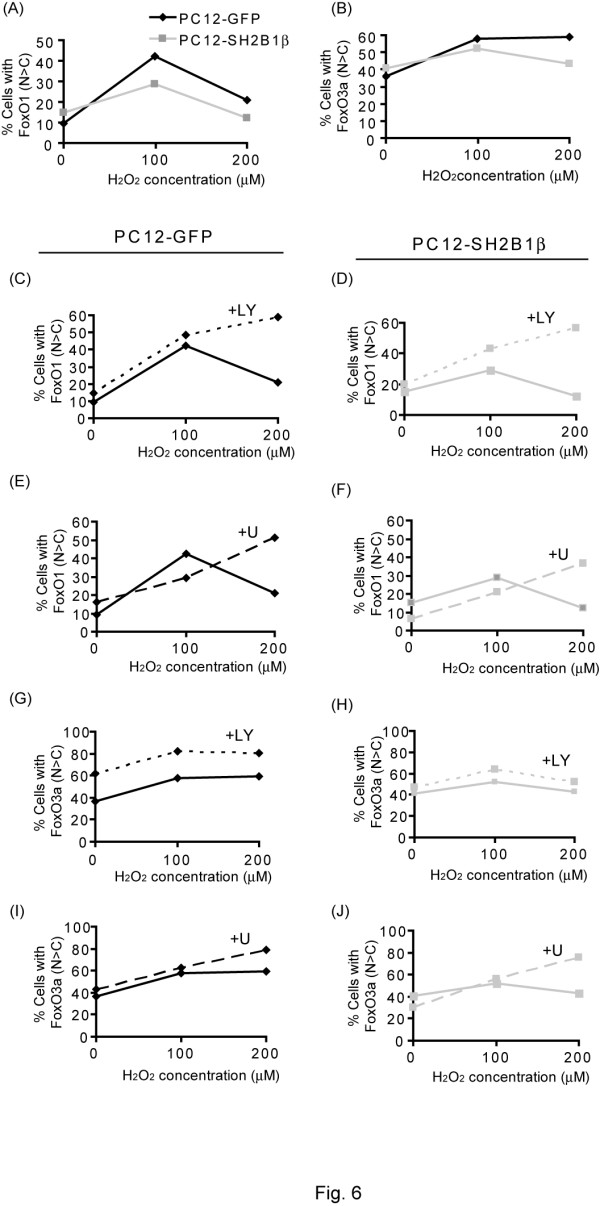
**SH2B1β reduces H_2_O_2_-induced nuclear distributions of FoxO1 and 3a**. PC12-GFP and PC12-SH2B1β cells were incubated in serum-free medium overnight before 0, 100, or 200 μM H_2_O_2 _treatment for 10 min, without (A-B) or with 20 μM LY294002 (+LY) (C-D, G-H) or 20 μM U0126 (+U) (E-F, I-J) pretreatment for 30 min. The localization of FoxOs was determined via immunofluorescence staining using anti-FoxO1 (A, C, E, G, I) or anti-FoxO3a (B, D, F, H, J) antibody followed by Alexa Fluor 555-conjugated secondary antibody. Images were taken using inverted Zeiss Axiover 135 fluorescence microscope. Percentage of cells with fluorescence intensity of FoxO1 or FoxO3a in the nucleus higher than in the cytoplasm (N > C) was quantified. A total of 90-110 cells were counted for each condition. For inhibitor assays, results from PC12-GFP cells are shown on the left panels and those from PC12-SH2B1β cells are shown on the right.

To examine the effect of SH2B1β on the distribution of FoxOs, PC12-GFP and PC12-SH2B1β cells were treated with H_2_O_2 _and the localization of FoxO1 and 3a were determined via immunofluorescence staining. The percentage of cells with FoxO1 fluorescence intensity in the nucleus higher than that in the cytoplasm was quantified and compared between the two stable cell lines. As expected, H_2_O_2 _increased nuclear localization of FoxO1 in both cell lines. Overexpressing SH2B1β reduced nuclear localization of FoxO1 by 15% and 8% in response to 100 and 200 μM H_2_O_2 _respectively (Figure 6A). In contrast, SH2B1β reduced nuclear localization of FoxO3a by 6% and 16% in response to 100 and 200 μM H_2_O_2 _(Figure 6B). Because pAKT and pERK1/2 were induced by different concentration of H_2_O_2_, the contribution of these signaling pathways to FoxO distribution was determined through inhibitor assays. In PC12-GFP cells, H_2_O_2_-induced nuclear distribution of FoxO1 was increased in the presence of PI3K and MEK inhibitors (LY294002 and U0126 respectively), suggesting the involvement of pAKT and pERK1/2 in cellular distribution of FoxO1 (Figure 6C, E). In PC12-SH2B1β cells, inhibiting PI3K increased nuclear localization of FoxO1 when treated with 100 and 200 μM H_2_O_2_, while inhibiting MEK increased the nuclear localization of FoxO1 at 200 μM H_2_O_2 _(Figure 6D, F). The effect of PI3K inhibitor on FoxO1 localization in PC12-SH2B1β cells was much more significant than that in PC12-GFP cells suggesting that SH2B1β promotes the cytoplasmic distribution of FoxO1 largely through PI3K-AKT pathway.

**Figure 7:**
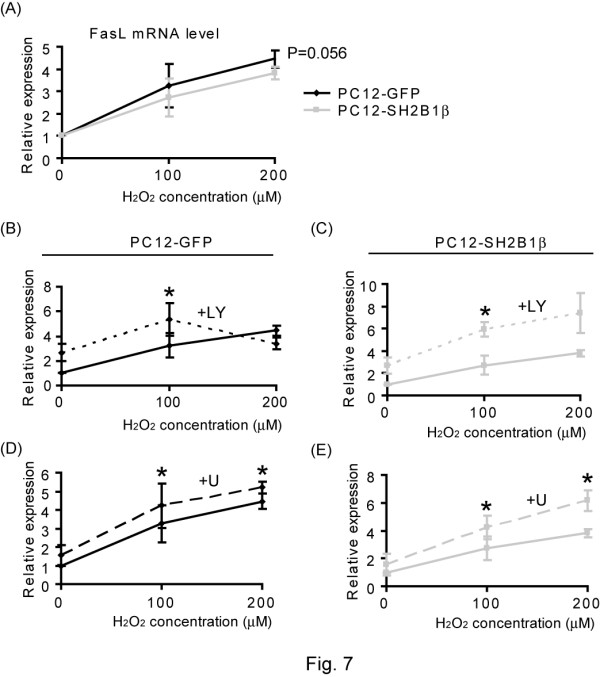
**SH2B1β reduces H_2_O_2_-induced expression of FasL through PI3K-AKT and MEK-ERK1/2**. PC12-GFP and PC12-SH2B1β cells were incubated in serum-free medium overnight, before H_2_O_2 _(0, 100, or 200 μM) treatment for 4 h without (A) or with 20 μM LY294002 (+LY) (B, C) or 20 μM U0126 (+U) (D, E) pre-treatment for 30 min. The expression of FasL was determined by Q-PCR. The relative levels of FasL were normalized to the expression of GAPDH. The relative expression levels of H_2_O_2_-treated samples were normalized to untreated samples in each cell line for each experiment. Values are expressed as mean ± S.E. from three independent experiments and statistically compared using student's *t*-test (*, P < 0.05). For inhibitor assays, results from PC12-GFP cells are shown on the left panels and those from PC12-SH2B1β cells are shown on the right.

**Figure 8:**
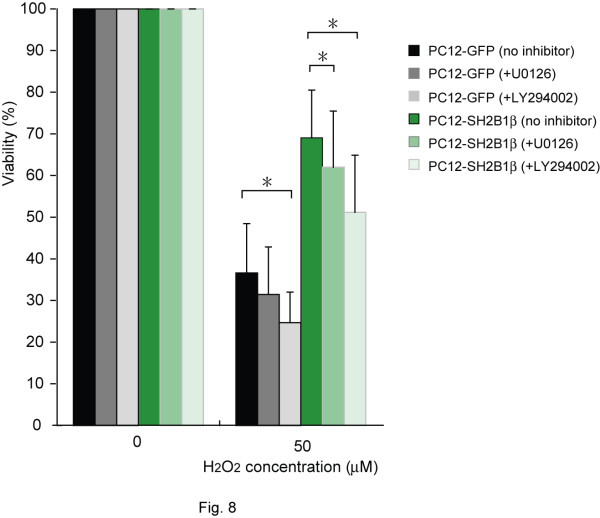
**Activation of PI3K-AKT and MEK-ERK1/2 signaling pathways contributes to cell survival in response to H_2_O_2_**. PC12-GFP or PC12-SH2B1β cells were pre-treated with PI3K inhibitor (LY294002), MEK inhibitor (U0126) or mock-treated 1 h before the addition of H_2_O_2 _for 24 h. Cell viability was measured by MTT assays. Values are expressed as % of the respective controls (samples without H_2_O_2 _treatment) for each experiment, with the values obtained from respective control cells taken as 100%. Values are expressed as mean ± S.E. from five independent experiments. The asterisks represent significant differences compared between the indicated conditions (*, p < 0.05).

For FoxO3a distribution, inhibiting PI3K increased its nuclear localization for both cell lines whereas inhibiting MEK increased its nuclear localization when treated with 200 μM H_2_O_2 _(Figure 6G, H, I, J). The effect of MEK inhibitor on the nuclear localization of FoxO3a was more prominent in PC12-SH2B1β cells than that in PC12-GFP cells suggesting that SH2B1β may increase pERK1/2 to regulate the distribution of FoxO3a in response to 200 μM H_2_O_2_. To determine whether SH2B1β regulates the transcriptional activity of FoxOs, the expressions of FasL were assessed via semi-quantitative real time polymerase chain reaction (Q-PCR). As in Figure 7A, the expression of FasL was induced in response to H_2_O_2 _treatment and the induction was reduced when SH2B1β was overexpressed. Inhibiting PI3K using LY294002 significantly increased the expression of FasL for both cell lines in response to 100 μM H_2_O_2 _treatment (Figure 7B, C). The extent of increase was more pronounced in PC12-SH2B1β cells than in PC12-GFP cells. Inhibiting MEK using U0126 significantly increased the expression of FasL for both cell lines in response to 100 as well as 200 μM H_2_O_2 _stimulation (Figure 7D, E). Similarly, the increase of FasL expression was more in PC12-SH2B1β cells than that in PC12-GFP cells. These results suggest that overexpressing SH2B1β enhances H_2_O_2_-induced PI3K-AKT and MEK-ERK1/2 signaling, leading to reduced nuclear localization of FoxO3a, and thus the reduction of FasL expression. To examine the contribution of PI3K-AKT and MEK-ERK1/2 signaling to SH2B1β-mediated cell survival, MTT assays were performed. As in Figure 8, inhibiting PI3K or MEK reduced cell viability by 5-10% in PC12-GFP cells and by 10-15% in PC12-SH2B1β cells for each inhibitor. These results suggest that both PI3K-AKT and MEK-ERK1/2 signaling contributes to SH2B1β-mediated cell survival.

Taken together, results from this study suggest that the adaptor protein SH2B1β reduces H_2_O_2_-induced apoptosis in PC12 cells and hippocampal neurons. SH2B1β protects cells in part through enhancing H_2_O_2_-induced phosphorylation of AKT and ERK1/2, reducing the nuclear localization of FoxOs and thus reducing the expression of a pro-apoptotic gene, FasL. This is the first demonstration that the adaptor protein SH2B1β reduces H_2_O_2_-induced and caspase 3-dependent apoptosis.

## Discussion

SH2B1 has been implicated in neuronal differentiation, cell growth, metabolism, obesity and diabetes [39,41,65-68]. Its ability to modulate cellular signaling confers its ability to regulate diverse functions. The only evidence so far that directly demonstrates its importance in cell survival is a study by Qian *et al *[67]. Injecting anti-SH2B1 antibody to sympathetic neurons leads to cell death suggesting that SH2B1 is required for neuronal survival [67]. However, it is not known how SH2B1 may influence live and death decision of cells. In the present study, we demonstrated that overexpressing SH2B1β reduced H_2_O_2_-induced cell death in PC12 cells and hippocampal neurons. In addition, overexpressing SH2B1β enhanced PI3K-AKT and MEK-ERK1/2 survival pathways in response to H_2_O_2_. Consistent with what Davila D *et al *have shown, phosphorylation of AKT was reduced as the concentration of H_2_O_2 _increased [22]. This reduction of pAKT may result from oxidation of plasma membrane and inactivation of surface receptors [69]. As oxidative stress increases, intracellular phosphatase, such as PP2A, is inhibited leading to the increase of pERK1/2 [21].

Overexpressing SH2B1β enhanced the phosphorylation of AKT and ERK1/2 which reduced the nuclear localization of FoxOs and FasL expression. Along this line, various reports also suggest the involvement of PI3K-AKT in promoting cell survival in hippocampal neurons [70-73] and our data suggest that SH2B1β-overexpressing neurons were not able to protect cells in the presence of PI3K inhibitor (data not shown). These results strongly implicate that SH2B1β protects neurons in part through PI3K-AKT pathway. In contrast, H_2_O_2 _slightly induced the expression of another FoxO-responsive gene --- MnSOD (Manganese Superoxide Dismutase) in PC12-GFP cells but the induction was much higher in PC12-SH2B1β cells (Additional file 4A). Furthermore, the expression of MnSOD was not significantly affected by either PI3K or MEK inhibitor (data not shown). Thus, SH2B1β may utilize PI3K-AKT- and MEK-ERK1/2-independent mechanisms to regulate the expression of MnSOD.

A report suggests that protein kinase D (PKD) triggers the activation of NFκB to increase MnSOD expression in response to oxidative stress [74]. However, we have not been able to detect H_2_O_2_-induced activation of NFκB. Accumulating evidence have demonstrated that the Janus tyrosine kinase (JAK)-Signal transduction and activators of transcription (STAT) signaling pathway plays an important role in the expression of stress-responsive genes as well as in cytoprotection in response to H_2_O_2 _[75,76]. A study also points to the involvement of STAT3 in MnSOD expression in response to hypoxia/reperfusion-induced injury and during liver regeneration [77,78]. Along the line, Stephanou *et al*. have shown that the JAK-STAT pathway participates in the modulation of expression of pro-survival Bcl2 proteins [79]. Interestingly, mRNA level of Bcl2 was found higher in PC12-SH2B1β cells compared to control cells (Additional file 4B). These findings suggest that SH2B1β may enhance the expression of survival genes through STAT3. The results from this study raise an intriguing possibility that the adaptor protein SH2B1β may utilize more than one mechanism to protect cells against stress and could act as a survival factor in general.

## Materials and methods

### Antibodies and reagents

MTT (3-(4,5-dimethylthiazol-2-yl)-2,5-diphenyltetrazolium bromide) was purchased from USB Corporation (Cleveland, OH). Hydrogen peroxide (H_2_O_2_), U0126 and LY294002 were from Calbiochem (San Diego, CA). Polyclonal antibody to rat SH2B1β was raised against a glutathione S-transferase fusion protein containing amino acids 527-670 of SH2B1β as described previously [32]. Whole antiserum against ERK1/2 was purchased form Sigma (St. Louis, MO). Mouse monoclonal antibodies to phospho-ERK1/2, phospho-S473 of AKT, rabbit polyclonal antibodies against AKT, phospho-FoxO1 (Ser256), FoxO1, FoxO3a and PARP were from Cell Signaling (Danvers, MA). Rabbit polyclonal antibody against phospho-FoxO3a/FKHRL1 (Thr32) was from Upstate (Temecula, CA). Anti-βIII tubulin (TUJ1) antibody was from Covance (Princeton, NJ). NGF, rat-tail collagen I, and growth factor-reduced Matrigel were purchased from BD Bioscience (Bradford, MA). Protein Assay Kit was purchased form Strong Biotech Corporation, Taiwan.

### Cell culture and microscopy

The stock of PC12 cells was purchased from American Type Culture Collection. PC12 cells were maintained on the collagen-coated plates (0.1 mg/ml) in complete media (DMEM supplemented with 10% heat-inactivated horse serum, 5% fetal bovine serum, 1 mM L-glutamine and 1 mM antibiotic-antimycotic). PC12 cells stably overexpressing GFP (PC12-GFP cells) or GFP-SH2B1β (PC12-SH2B1β cells) were made and cultured as described in Chen *et al *[65]. Pooled population was used to avoid clonal variation. The serum-free medium used was DMEM supplemented with 1% BSA, 1 mM L-glutamine and 1 mM antibiotic-antimycotic. For immunofluorescence staining, PC12-GFP and PC12-SH2B1β cells were treated with H_2_O_2 _for 10 min, then fixed, permeabilized and incubated with the indicated antibodies. Fluorescent images were taken using inverted Zeiss Axiover 135 fluorescence microscope (400× magnification). For anti-active caspase 3 staining, digital images were captured using upright Fluorescent Microscope Zeiss/Axioskop 2 mot plus. The fluorescent pixel spatial orientation and pixel intensity were measured by AxioVision 4.8 software. Signal of active caspase-3 fluorescence was localized mostly to cell nucleus and its fluorescent intensity in the nucleus was quantified using AxioVision 4.8.

### MTT and inhibitor assays

Cells were plated at a density of 3 × 10^4 ^cells/well in the Matrigel-coated 96-well plates. After overnight incubation, cells were treated with freshly prepared H_2_O_2_. Cell viability was assayed by the reduction of MTT following the manufacture's instruction. Results are presented as percentage of the control using the absorbance of the control cells is 100%. For inhibitor assay, cells were pretreated with inhibitors (20 μM U0126 or 20 μM LY294002) for 1 h (for MTT assays) or 30 min prior to H_2_O_2 _treatment.

### H_2_O_2 _treatment and immunoblotting

Cells were incubated in serum-free medium overnight before H_2_O_2 _treatment. Cells were lysed using lysis buffer (RIPA) containing freshly added 1 mM Na_3_VO_4_, 1 mM phenylmethanesulphonylfluoride (PMSF), 10 ng/ml aprotinin and 10 ng/ml leupeptin. Protein concentration of each sample was determined by protein assay kit. Samples with equal amount of proteins were resolved using 8% SDS-PAGE followed by Western blotting with specific primary antibodies. The immunoblots were detected using either IRDye 700- or IRDye 800CW-conjugated IgG and an Odyssey Infrared Imaging System (LI-COR Biosciences, Lincoln, NE) or horseradish peroxidase-conjugated IgG and the ECL (enhanced chemiluminescence) system. Western blots results were quantified using NIH Image J software.

### Measurement of intracellular ROS levels

Dihydroethidium (DHE) was purchased from Invitrogen (Carlsband, CA), and used to measure the production of intracellular ROS. DHE shows a blue fluorescence in cell cytoplasm until oxidization to form red fluorescent-ethidium which is trapped in the nucleus by intercalating into DNA. ROS levels were analyzed in FACSCalibur flow cytometer (Becton Dickinson, CA). Fluorescence was detected by filter FL-3 (670 nm). Histograms of 10,000 events were analyzed and DHE fluorescence was evaluated by using the CellQuest software (Becton Dickinson).

### Preparation of rat hippocampal neurons and transient transfection

Primary hippocampal neuron cultures were prepared from Sprague-Dawley rats as described previously [80,81]. Briefly, cells were dissociated from hippocampus dissected from embryonic day 18 (E18) rat embryos by treatment with papain (10 U/ml). Dissociated cells were washed and suspended in MEM supplemented with 5% horse serum and 5% fetal calf serum. Neurons were then plated onto coverslips coated with poly-L-lysine, and cultured in neurobasal medium with B27 (containing additional 0.025 mM glutamate) on DIV (day *in vitro*) 1. On DIV 3, the cells were treated with 5 μM cytosine 1-β-D-arabinofuranoside (ARC) for 1 day to inhibit the growth of glial cells. Medium was replaced by half of the fresh neurobasal/B27 medium on DIV4 and twice a week thereafter. GFP, GFP-SH2B1β or GFP-SH2B1β(R555E) was transfected to neurons on DIV3 using the CaCl_2 _transfection kits from Promega (Madison, WI). Two days after transfection, neurons were treated with H_2_O_2 _as indicated.

### RNA preparation and semi-quantitative real-time PCR

TRIzol reagent was use to isolate total RNA form PC12 cells with or without treatment at the indicated time. Concentrations and A_260/280 _ratios of RNAs were measured using spectrophotometer (NanoDrop 1000, Themo). Total RNA of each sample was reverse transcribed into cDNA and the relative gene expressions of FasL and glyceraldehydes-3-phosphate dehydrogenase (GAPDH) were determined via semi-quantitative PCR (Q-PCR) assay using SYBR green master mix and the ABI7500 system. Primer sequences for each gene were designed using PrimerExpress software. Amplicons generated from each primer pair were between 50 to 100 bp. Loading of each sample was normalized with ROX dye. All readings were normalized to the expression of GAPDH. The forward primer for FasL is 5' CTGGTGGCTCTGGTTGGAAT3' and the reverse primer is 5' CTCACGGAGTTCTGCCAGTTC 3'. The forward primer for GAPDH is 5' ATGACTCTACCCACGGCAAGTT3' and the reverse primer is 5' TCCCATTCTCAGCCTTGACTGT 3'.

### Statistical analysis

Data were expressed as mean ± S.E., and significant differences were analyzed by Student's *t*-test. The results are considered significant when P < 0.05.

## Abbreviations

ERK: extracellular signal-regulated kinase; MAPK: mitogen-activated protein kinases; MnSOD: Manganese superoxide dismutase; MEK: MAPK/ERK kinase; PI3K: phosphatidylinositol 3-kinas; AKT: protein kinase B. FoxO: forkhead box O, forkhead members of the O class; PARP: poly (ADP-ribose) polymerase; FasL: Fas ligand.

## Competing interests

The authors declare that they have no competing interests.

## Authors' contributions

WCL performed signaling experiments, caspase 3 and PARP western blot analysis, immunofluorescence staining and quantification of FoxOs, and gene expression. CJC performed hippocampal neuron isolation, transient transfection, active caspase 3 staining and quantification, ROS detection, and Annexin V staining. HCH contributed to signaling experiments and MTT assays. HLH performed MTT assays. LC is the corresponding author that designed, coordinated all experiments, wrote the manuscript and formatted figures. All authors read and approved the final manuscript.

## Supplementary Material

Additional file 1 **Hydrogen peroxide induces death of hippocampal neurons**. Hippocampal neurons from E18 embryos were isolated as described in the Materials and Methods. Neurons were treated with the indicated concentrations of H_2_O_2 _for 18 h, then fixed for immunofluorescence staining using anti-βIII tubulin (neuronal tubulin) antibody (shown in green) and DAPI (shown in blue). Click here for file

Additional file 2 **Overexpressing SH2B1β reduces H_2_O_2_-induced levels of Annexin V**. PC12-GFP and PC12-SH2B1β cells were treated with 0, 100 or 200 μM H_2_O_2 _for 18 h. The levels of Annexin V were quantified through flow cytometry. Click here for file

Additional file 3 **SH2B1β reduces and SH2B1β(R555E) increases H_2_O_2_-induced levels of active caspase 3 in hippocampal neurons**. Hippocampal neurons from E18 embryos were transiently transfected with GFP, GFP-SH2B1β or GFP-SH2B1β(R555E) on DIV 3 and then treated with H_2_O_2 _on DIV 5 for 18 h. Cells were fixed and subjected to immunofluorescence staining using anti-active caspase 3 antibody (shown in red) and DAPI (shown in blue). Fluorescence intensity was quantified using AxioVision 4.8 (Zeiss) and shown in the bottom panel. Click here for file

Additional file 4 **Overexpressing SH2B1β increases the gene expressions of MnSOD and Bcl2**. PC12-GFP and PC12-SH2B1β cells were treated with 0, 100 or 200 μM H_2_O_2 _for 4 h. Total RNAs were extracted and subjected to Q-PCR analysis. (A) Primers for MnSOD: forward 5' ATTAACGCGCAGATCATGCAG 3'; reverse 5' TTTCAGATAGTCAGGTCTGACGTT 3'. (B) Primers for Bcl2: forward 5' TGGGATGCCTTTGTGGAACT 3'; reverse 5' CAGCCAGGAGAAATCAAACAGA 3'. Data were normalized to GAPDH and untreated PC12-GFP samples. Click here for file
