# Supplementary material for: The adaptor protein SH2B1β reduces hydrogen peroxide-induced cell death in PC12 cells and hippocampal neurons
Source: J Mol Signal. 2010 Sep 27;5:17. doi: 10.1186/1750-2187-5-17 (PMC2954984; doi:10.1186/1750-2187-5-17)
Supplement: Additional file 1 — Hydrogen peroxide induces death of hippocampal neurons. Hippocampal neurons from E18 embryos were isolated as described in the Materials and Methods. Neurons were treated with the indicated concentrations of H2O2 for 18 h, then fixed for immunofluorescence staining using anti-βIII tubulin (neuronal tubulin) antibody (shown in green) and DAPI (shown in blue). [file 1750-2187-5-17-S1.PDF]

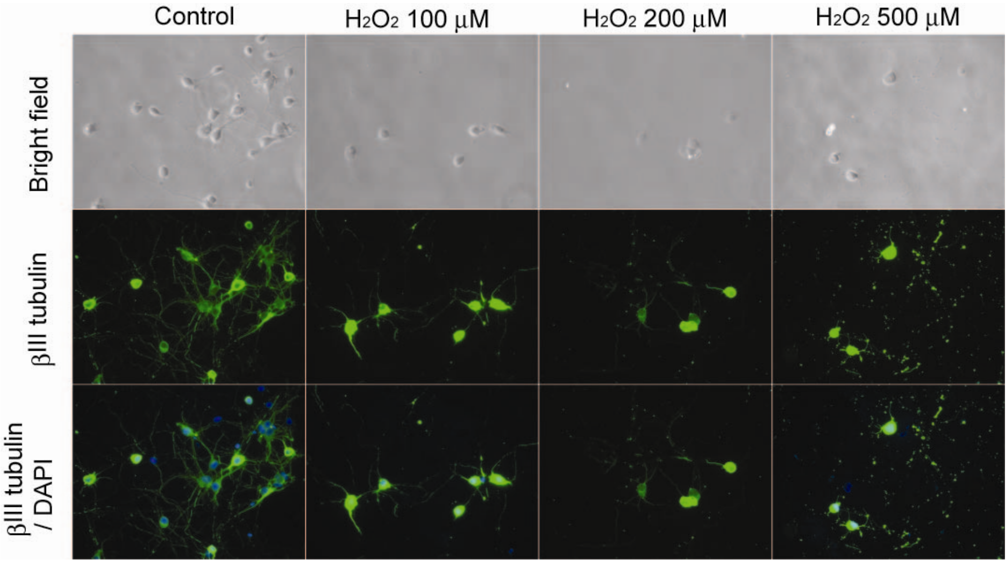

Additional file 1: Hydrogen peroxide induces death of hippocampal neurons.

Hippocampal neurons from E18 embryos were isolated as described in the Materials and Methods. Neurons were treated with the indicated concentrations of H_2O_2 for 18 h, then fixed for immunofluorescence staining using anti-βIII tubulin (neuronal tubulin) antibody (shown in green) and DAPI (shown in blue).
