# Supplementary material for: The adaptor protein SH2B1β reduces hydrogen peroxide-induced cell death in PC12 cells and hippocampal neurons
Source: J Mol Signal. 2010 Sep 27;5:17. doi: 10.1186/1750-2187-5-17 (PMC2954984; doi:10.1186/1750-2187-5-17)
Supplement: Additional file 2 — Overexpressing SH2B1β reduces H2O2-induced levels of Annexin V. PC12-GFP and PC12-SH2B1β cells were treated with 0, 100 or 200 μM H2O2 for 18 h. The levels of Annexin V were quantified through flow cytometry. [file 1750-2187-5-17-S2.PDF]

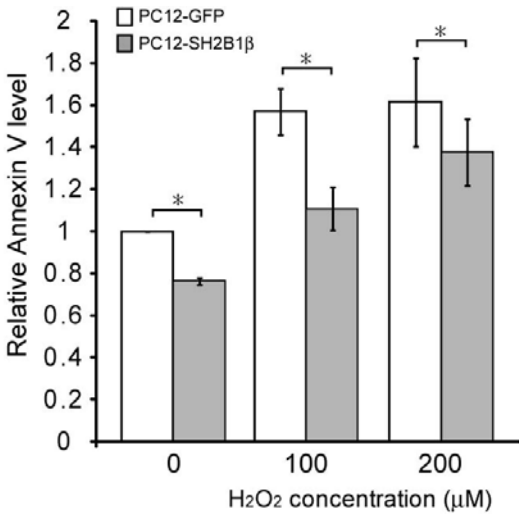

Additional file 2: Overexpressing SH2B1β reduces H_2O_2–induced levels of Annexin V.

PC12-GFP and PC12-SH2B1β cells were treated with 0, 100 or 200 μM H_2O_2 for 18 h. The levels of Annexin V were quantified through flow cytometry. *: $p<0.05$.
