# Supplementary material for: The adaptor protein SH2B1β reduces hydrogen peroxide-induced cell death in PC12 cells and hippocampal neurons
Source: J Mol Signal. 2010 Sep 27;5:17. doi: 10.1186/1750-2187-5-17 (PMC2954984; doi:10.1186/1750-2187-5-17)
Supplement: Additional file 3 — SH2B1β reduces and SH2B1β(R555E) increases H2O2-induced levels of active caspase 3 in hippocampal neurons. Hippocampal neurons from E18 embryos were transiently transfected with GFP, GFP-SH2B1β or GFP-SH2B1β(R555E) on DIV 3 and then treated with H2O2 on DIV 5 for 18 h. Cells were fixed and subjected to immunofluorescence staining using anti-active caspase 3 antibody (shown in red) and DAPI (shown in blue). Fluorescence intensity was quantified using AxioVision 4.8 (Zeiss) and shown in the bottom panel. [file 1750-2187-5-17-S3.PDF]

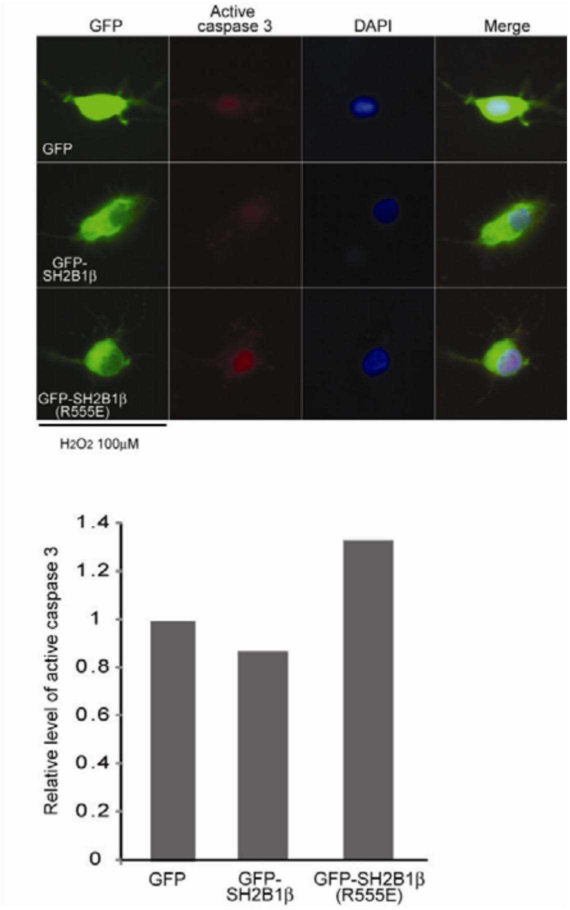

Additional file 3: SH2B1β reduces and SH2B1β(R555E) increases H_2O_2–induced levels of active caspase 3 in hippocampal neurons.

Hippocampal neurons from E18 embryos were transiently transfected with GFP, GFP-SH2B1β or GFP-SH2B1β(R555E) on DIV 3 and then treated with H_2O_2 on DIV 5 for 18 h. Cells were fixed and subjected to immunofluorescence staining using anti-active caspase 3 antibody (shown in red) and DAPI (shown in blue). Fluorescence intensity was quantified using AxioVision 4.8 (Zeiss) and shown in the bottom panel.
