# Supplementary material for: The adaptor protein SH2B1β reduces hydrogen peroxide-induced cell death in PC12 cells and hippocampal neurons
Source: J Mol Signal. 2010 Sep 27;5:17. doi: 10.1186/1750-2187-5-17 (PMC2954984; doi:10.1186/1750-2187-5-17)
Supplement: Additional file 4 — Overexpressing SH2B1β increases the gene expressions of MnSOD and Bcl2. PC12-GFP and PC12-SH2B1β cells were treated with 0, 100 or 200 μM H2O2 for 4 h. Total RNAs were extracted and subjected to Q-PCR analysis. (A) Primers for MnSOD: forward 5' ATTAACGCGCAGATCATGCAG 3'; reverse 5' TTTCAGATAGTCAGGTCTGACGTT 3'. (B) Primers for Bcl2: forward 5' TGGGATGCCTTTGTGGAACT 3'; reverse 5' CAGCCAGGAGAAATCAAACAGA 3'. Data were normalized to GAPDH and untreated PC12-GFP samples. [file 1750-2187-5-17-S4.PDF]

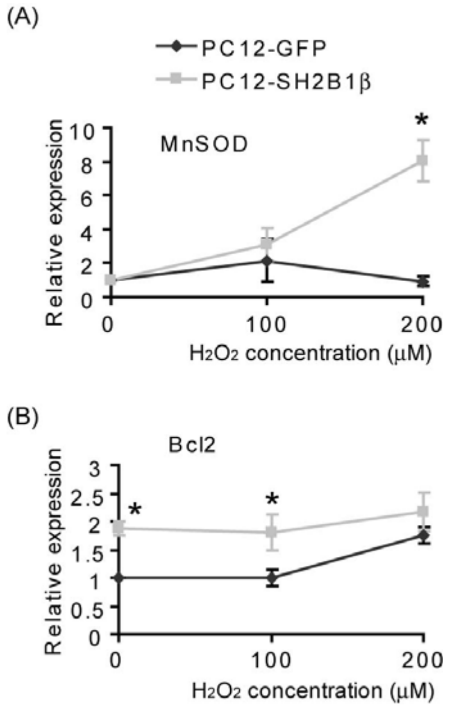

Additional file 4: Overexpressing SH2B1β increases the gene expressions of MnSOD and Bcl2.

PC12-GFP and PC12-SH2B1β cells were treated with 0, 100 or 200 μM H_2O_2 for 4 h. Total RNAs were extracted and subjected to Q-PCR analysis. (A) Primers for MnSOD: forward 5’ ATTAACGCGCAGATCATGCAG 3’; reverse 5’ TTTCAGATAGTCAGGTCTGACGTT 3’. (B) Primers for Bcl2: forward 5’ TGGGATGCCTTTGTGGAACT 3’; reverse 5’ CAGCCAGGAGAAATCAAACAGA 3’. Data were normalized to GAPDH and untreated PC12-GFP samples. *: $p<0.05$.
